# Theoretically-Based Emotion Regulation Strategies Using a Mobile App and Wearable Sensor Among Homeless Adolescent Mothers: Acceptability and Feasibility Study

**DOI:** 10.2196/pediatrics.9037

**Published:** 2018-01-03

**Authors:** Noelle R Leonard, Bethany Casarjian, Richard R Fletcher, Cathleen Praia, Dawa Sherpa, Anna Kelemen, Sonali Rajan, Rasheeda Salaam, Charles M Cleland, Marya Viorst Gwadz

**Affiliations:** 1 Center for Drug Use and HIV Research, Rory Meyers College Of Nursing, New York University, New York, NY, United States; 2 Teachers College, Columbia University, New York, NY, United States; 3 Lionheart Foundation, Boston, MA, United States; 4 MIT Media Lab, Cambridge, MA, United States; 5 Independent Consultant, Boston, MA, United States

**Keywords:** electrodermal response, adolescence, mothers, emotion, parenting

## Abstract

**Background:**

Many adolescent mothers are parenting young children under highly stressful conditions as they are managing first-time parenthood, poverty, lack of housing, school and work, and challenging peer and familial relationships. Mobile health (mHealth) technology has the potential to intervene at various points in the emotion regulation process of adolescent mothers to provide them support for more adaptive emotional and behavioral regulation in the course of their daily life.

**Objective:**

The goal of this study was to examine the acceptability, feasibility, use patterns, and mechanisms by which a mobile technology used as an adjunct to in-person, provider-delivered sessions fostered adolescent mothers’ adaptive emotion regulation strategies under real-life conditions.

**Methods:**

Participants (N=49) were enrolled in the intervention condition of a larger pilot study of homeless adolescent mothers living in group-based shelters. The mHealth technology. Calm Mom, consisted of a mobile app and a wrist-worn sensorband for the ambulatory measurement and alerting of increased electrodermal activity (EDA), a physiological measurement of stress. We examined logs of mobile app activity and conducted semistructured qualitative interviews with a subsample (N=10) of participants. Qualitative data analysis was guided by the theoretical frames of the intervention and a technology acceptance model and included an analysis of emerging themes and concepts.

**Results:**

Overall, participants indicated that one or more of the elements of Calm Mom supported their ability to effectively regulate their emotions in the course of their daily life in ways that were consonant with the intervention’s theoretical model. For many adolescent mothers, the app became an integral tool for managing stress. Due to technical challenges, fewer participants received sensorband alerts; however, those who received alerts reported high levels of acceptability as the technology helped them to identify their emotions and supported them in engaging in more adaptive behaviors during real-life stressful situations with their children, peers, and family members.

**Conclusions:**

Calm Mom is a promising technology for providing theoretically driven behavioral intervention strategies during real-life stressful moments among a highly vulnerable population. Future research efforts will involve addressing technology challenges and refining tailoring algorithms for implementation in larger-scale studies.

## Introduction

### Background

Adolescent mothers often experience tremendous stress managing the concurrent developmental transitions of adolescence and new motherhood. A vast majority of adolescent mothers in the United States are between the ages of 15 and 19 years [1] and are single parents balancing new motherhood, school, and work while navigating the normative social and emotional challenges of adolescence [2]. Although they experience stress typical of new mothers [3–5], many adolescent mothers enter parenthood with histories of adverse childhood events including maltreatment, trauma, foster care placement, and homelessness in addition to engagement in sexual and other risk behaviors including substance use and delinquency [6,7]. Adolescent mothers’ parenting practices may be compromised by these early disadvantages and current demands, placing them at great risk for neglectful and abusive parenting [3,8].

Managing or regulating emotions is a core developmental skill that develops through childhood and adolescence and is critical for both effective parenting and successfully navigating the transition from adolescence to early adulthood [9–11]. Emotion regulation is defined as the process by which individuals modify the expression and experience of their emotions in ways that are sensitive to situational demands [12]. Effective emotion regulatory abilities among parents are associated with sensitive, positive parenting and better parental mental health, whereas poor emotion regulation places parents at higher risk for child maltreatment [13]. Compared with adult mothers, adolescent mothers are more likely to experience decreased tolerance of children’s negative affect and have difficulty engaging in sensitive and empathic parenting [14,15]. Moreover, those with adverse childhood experiences often have a heightened sensitivity to stressful events [16]. Increasing emotional regulatory strategies has the potential to assist adolescent mothers in navigating these transitions and engaging in more effective parenting.

Behavioral interventions targeting mental health issues such as stress can be delivered in the real world, in real time, and *just-in-time*, when individuals actually need support via mobile phones and other technologies [17–19]. Mobile health (mHealth) interventions that adapt to changes in individuals’ internal physiological states have the potential to provide personalized support at specific vulnerable periods. To date, the vast majority of adaptive behavioral interventions use wearable, ambulatory technologies that continuously monitor activity level through accelerometers (eg, [20]); fewer have used physiological measures of stress.

Electrodermal activity (EDA) exclusively measures sympathetic nervous system activity, whose general action is to mobilize the body’s response to emotional arousal [21]. Under relatively low levels of physical exertion, higher EDA reflects emotional arousal, particularly stress, including arousal not open to conscious awareness, as well as attention-demanding tasks such as parenting [22]. Traditionally, EDA has been measured in the laboratory under simulated emotionally arousing conditions, but recently, validated, wearable, and wireless methods of ambulatory monitoring of EDA have been developed for use in everyday life [23–25], and they hold great promise for assisting individuals to recognize and regulate strong emotions. However, outside of small, proof-of-concept studies conducted over brief periods of time [23,24,26,27], few studies have examined the continuous, longitudinal use of wearable sensors for monitoring EDA under real-life conditions.

In this report, we examine the feasibility and acceptability of an mHealth technology that consists of a mobile app and wearable sensorband to measure and alert participants of increased EDA. The technology was used as an adjunct to an in-person, provider-delivered intervention for increasing emotion regulation and positive parenting and reducing risk behaviors among homeless adolescent mothers.

### Emotion Regulation

The extended process model of emotion regulation (EPMER) [12] is a theoretical framework that delineates a sequence of 3 dynamic and recursive steps in the emotional regulatory process: *identification*, *selection*, and *implementation*. Each step represents a potential target for regulation. In the *identification* stage, individuals detect the emotion and valence (ie, largely positive or negative). In the *selection* stage, individuals determine a strategy for regulating the emotion, including the following: *situation selection* and *modification*, which entail selecting or changing the emotion-generating environment or context; *attentional deployment*, which includes directing or shifting one’s attention to influence the emotion; *cognitive change*, which entails altering one’s cognitive appraisal of the situation to influence the emotional impact; and *response modulation*, which entails directly influencing the behavioral or physiological aspects of the emotional response. Finally, in the third stage, the regulation strategy is *implemented* in ways that are appropriate to the context. The effective regulation of emotions increases the probability that individuals will enact more adaptive behaviors [28].

### Strategies for Increasing Emotion Regulation: Cognitive Behavioral, Mindfulness Meditation, and Mindful Parenting

Cognitive behavioral therapy (CBT) [29,30] and mindfulness meditation [31–33] have been found to work synergistically to increase adaptive emotional regulation at various stages in the emotion regulation process. CBT-derived techniques include both cognitive and behaviorally focused strategies for examining and reappraising automatic thoughts and assumptions and building stress reduction and coping skills for dealing with situation-specific stressors. Mindfulness meditation involves training to increase mindful awareness of moment-by-moment experiences in a nonjudgmental, accepting manner [31]. The cultivation of mindfulness has been demonstrated to increase cognitive and emotion regulatory skills among adolescents, most notably, attention regulation and inhibitory control [34,35], and decrease negative emotionality [36,37]. Moreover, strategies to increase mindfulness in parenting have been found to increase parents’ emotional awareness and self-regulation in parent-child interactions; reduce automatic, negative reactions toward children; and increase parental sensitivity [38,39] Receiving physiological feedback about emotional states can add potency to CBT and mindfulness meditation strategies.

### Applying Intervention Strategies to Interactive Digital Technologies

Despite the proliferation of mHealth technologies, theories of technology-based behavior change have only emerged recently [40–43]. The effective translation of behavioral interventions to interactive digital technologies may be highly dependent upon the fit between the target behaviors, the specific theoretically grounded behavior change strategies employed in the technology, and the relationship between the functional aspects of the technology and user preferences.

Finally, blended interventions that incorporate both provider-delivered intervention and mHealth technology are less frequently reported, yet have great potential to assist individuals to practice skills learned with providers in the context of their natural environment. Some initial evidence suggests that blended interventions may be more efficacious for reducing stress compared with technology-only interventions [44], especially among adolescents [45].

Thus, in a real-world study of homeless adolescent mothers, we used quantitative and qualitative data to examine (1) participants’ engagement with the technology components of the intervention and (2) the ways participants experienced the theoretically based emotional and behavioral regulatory strategies through the technology component. Specifically, we were interested in understanding the mechanisms by which the technology components fostered emotional regulation strategies.

## Methods

### Participants

Participants in this study were enrolled in the intervention condition (N=49 adolescent mothers) of a pilot randomized controlled trial for homeless adolescent mothers living with their children in transitional living programs (TLPs) in a northeast state. The description and results of the randomized controlled study are not reported in this manuscript but are forthcoming. TLPs are large group home shelters that are staffed 24 horns per 7 days a week and house between 8 and 20 adolescent mothers and their children. Adolescent mothers and their children are eligible for voluntary shelter in the TLPs if the adolescent mothers are aged between 13 and 21 years, homeless, and eligible for public assistance, and have custody of their children [46]. Adolescent mothers typically reside in the TLP with their children for 6 to 8 months, although the lengths of stay vary widely.

Between 2013 and 2015, adolescent mothers participated in a baseline interview, which included survey items of demographic/background characteristics and an assessment of EDA (described below) and received a US $40 stipend. The in-person intervention was conducted weekly in a group format for 1.5 hours in the common room of the TLR Participants received a study smartphone (typically during session 1) and a sensorband (typically during session 2). Adolescent mothers also participated in 2 follow-up interviews at 3 and 6 months post baseline and received a $25 stipend for each interview. The study phone was provided for the duration of the study (6 months) and the sensorband was provided for the first 3 months (study flow chart. Multimedia Appendix 1). Participants who were emancipated minors or who were 18 years old or older provided informed consent for participation in all study activities. Informed consent for participants under 18 years was provided by the participants’ caseworker or parent/legal guardian (if available), and these adolescent mothers also provided informed assent for participation. The New York University’s institutional review board (IRB) and the IRB of the state’s child welfare agency approved all aspects of the study.

To examine participants’ experiences with the technology, a subsample of adolescent mothers who used one or more components on at least one occasion were randomly selected from each of the TLPs to participate in an in-depth, semistructured qualitative interview after their 3-month follow-up assessment. Of the 21 available participants, we conducted interviews with 10 participants who received $25 for their participation.

### Description of the Blended Intervention Developed for This Study

Power Source Parenting is a theoretically based parenting intervention aimed at highly vulnerable adolescent mothers and delivered by trained interventionists [47]. Calm Mom is the companion mHealth technology consisting of a smartphone app developed for the study and an integrated wearable sensorband that continually measures EDA [23,48]. The Calm Mom technology is designed to reinforce the emotional regulatory and positive parenting skills learned in the Power Source Parenting in-person sessions by delivering intervention material, just-in-time, when adolescent mothers need to support to enact these skills in between Power Source Parenting sessions.

Intervention techniques in the Power Source Parenting in-person sessions involve CBT and mindfulness meditation strategies aimed at increasing positive child management and mindful caretaking skills, increasing knowledge of nonnative child development, and providing strategies for dealing with stress, risk behaviors (eg, substance use, delinquency), and challenging relationships with peers, family members, romantic/sexual partners, and their child’s father. The manualized intervention consists of interactive group exercises, discussions, role plays, brief videos, and outside readings [47], as well as formal sitting meditation focusing on the breath and body awareness.

**Figure 1.**
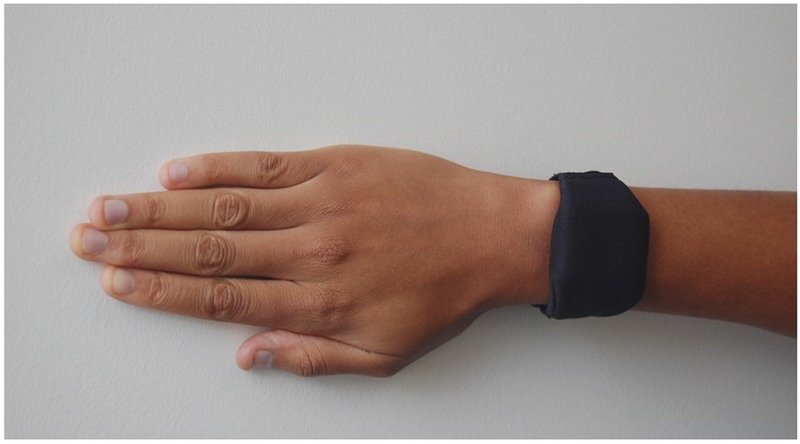
Calm Mom sensorband

Calm Mom consists of a mobile app developed for the study and a biosensorband (see Figure 1). The sensorband is worn on the wrist or ankle and continuously measures EDA, sending this wirelessly via Bluetooth [49] to an Android-based smartphone (technical specifics and cost have been described elsewhere) [23,24,48]. Pilot testing of the sensorband was conducted with a small number of participants in the target population before initiation of this study [48].

**Figure 2.**
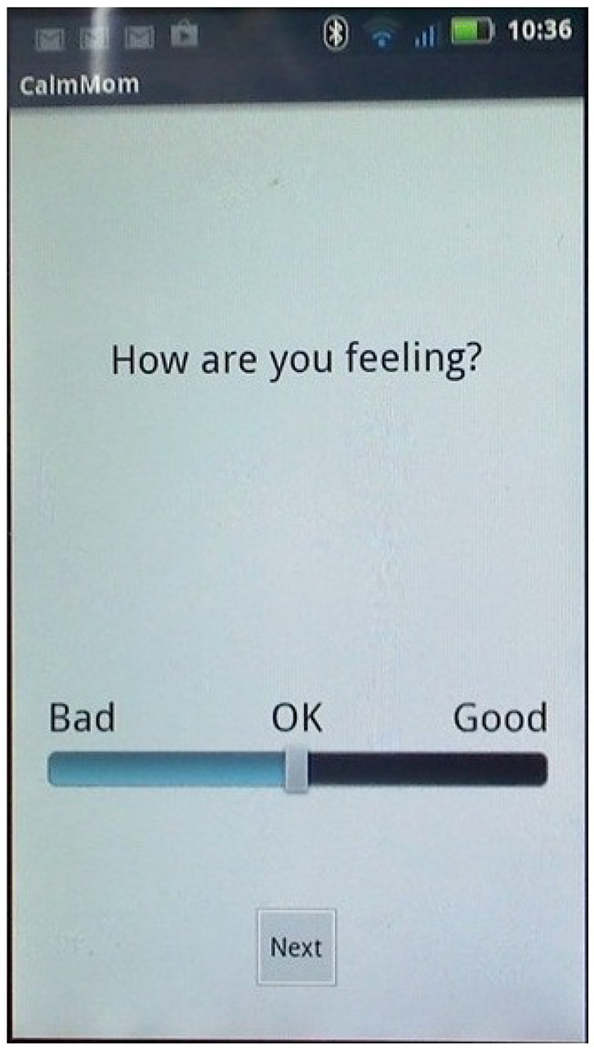
Rating feelings screenshot.

**Figure 3.**
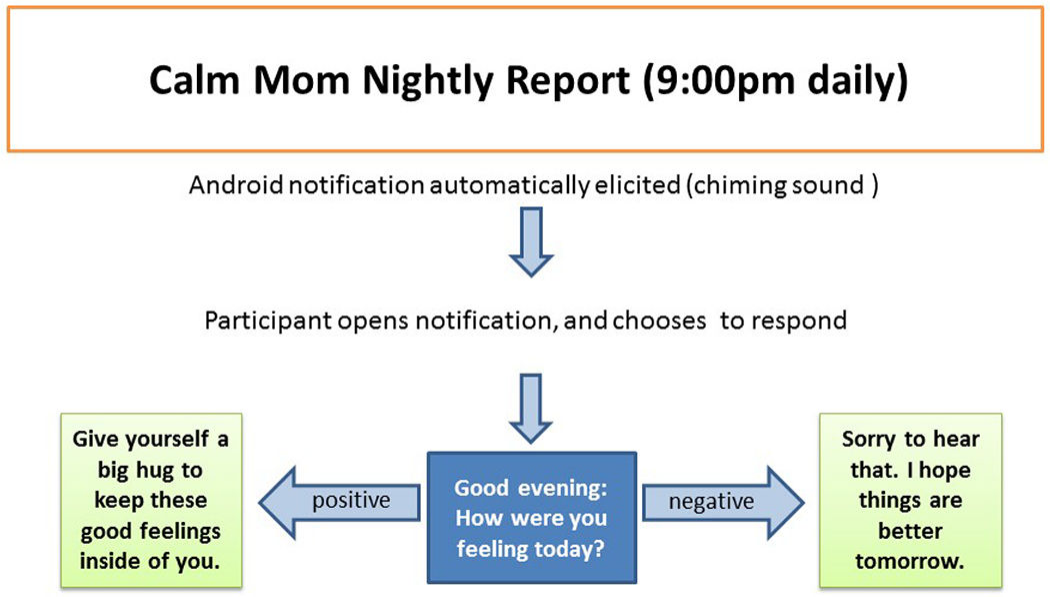
Flowchart for nightly report.

In an effort to provide a variety of features. Calm Mom elements were delivered to participants in 3 different ways, using both push-in (notifications and requests are sent by the system) and pull (requests are made by the user) designs. Of the 3 elements, 2 were delivered independent of the sensorband via the mobile app alone: (1) a nightly report alert delivered at 9 PM every evening (push-in) and (2) a self-report, which participants were free to initiate at any time (pull). The mobile app combined with the sensorband elicited a sensorband-triggered alert that signaled the participant when her EDA reached an individually determined threshold and invited her to make a report on the app (push-in). Each element began with a screen on the app that asked, “Would you like to make a report?” If participants answered “no,” the app closed. If they answered “yes,” they were asked to rate their feelings using a slide bar (see Figure 2), using a scale of 0 (“Bad”) to 100 (“Good”), with neutral (50: “Okay”) as the default (50 and above is considered within the positive range). As seen in Figure 3, the nightly report was brief and consisted of an affirmational message congruent with the valence.

**Figure 4.**
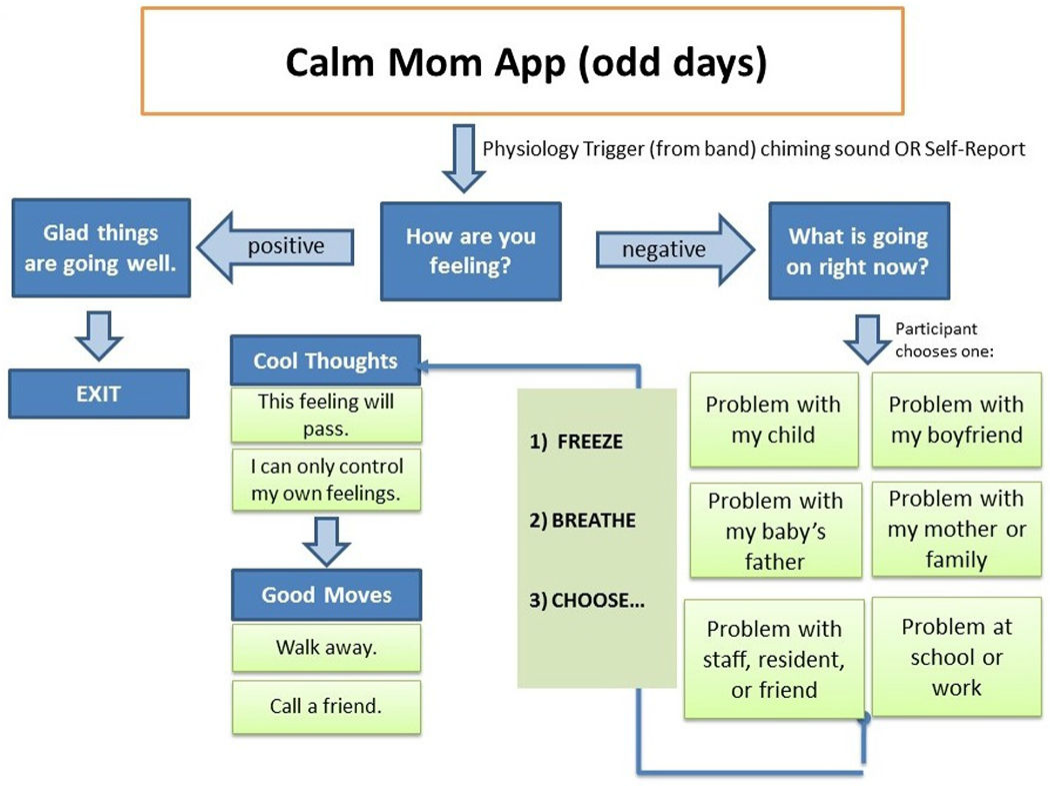
Flowchart for sensor-triggered or self-report on odd days.

**Figure 5.**
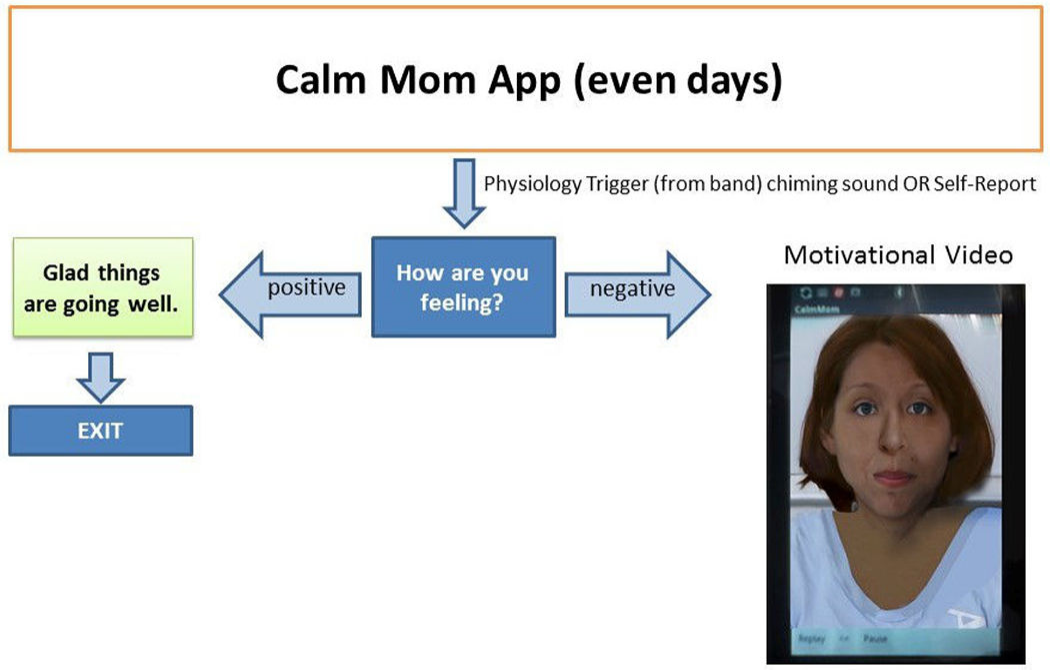
Flowchart for sensor-triggered or self-report on even days.

Self-reports and sensor-triggered alerts involved more content on the app, and in an effort to minimize participant burden, we alternated the length of reporting for the sensorband-triggered alert and self-report on odd (Figure 4) and even days (Figure 5). Specifically, on odd days, participants were asked to rate their feelings on a slide bar (as described above). If the valence was in the positive range, an affirmational message popped up.

If the valence selected was in the negative range, adolescent mothers would be asked to report the problem type via radio buttons from a list of 6 main problem areas that adolescent mothers typically encounter (eg, child, boyfriend, school/work) and read a motivational/coping message (eg, “No matter how you’re feeling right now, remember that you love your baby”) and a behavioral skill (eg, “Freeze, breathe, and choose”) previously learned in the in-person sessions and related to the problem type chosen. Alternatively, on even days, adolescent mothers were asked to rate their feelings as above and then watch a brief (<20 sec) video of inspirational and behavioral messages congruent with the valence reported delivered by ethnically/racially diverse adolescent mothers. Both nightly reports and sensorband-triggered alerts provided a “snooze alarm” period, enabling mothers to put the alert on hold for up to two 10-min periods.

### Measures and Data Sources

An assessment of baseline EDA was conducted to determine participants’ individual EDA threshold using a cognitive stress task and physical activity. Participants were asked to sit quietly in a comfortable position for a few moments, and after signing informed consent/assent, the research assistant then placed the sensorband on the participant’s right wrist while she sat quietly for a few moments. Participants then engaged in a computerized Stroop color word task in which they were asked to read the names of the colors as quickly as possible in 1 min, and a brief physical task (eg, walking up and down the stairs quickly) was performed for approximately 4 min.

Data were obtained from 3 sources: (1) survey results conducted at the baseline interview of participants in the intervention arm for demographic information and a quantitative acceptability measure at the 3-month follow-up interview, (2) logs of the technology activity, and (3) semistructured qualitative interviews from the subsample of adolescent mothers who used the technology on at least one occasion.

The acceptability questionnaire consisted of 8 items developed for the study with the item wording and response categories derived from the client satisfaction questionnaire [50]. The questions asked about participants’ satisfaction of the blended intervention as a whole, satisfaction and usefulness of the mobile app and the sensorband for reducing their stress and dealing with their children, and the frequency to which participants used specific skills from the in-person intervention that also appeared on the mobile app (eg, “Freeze, breathe, and choose”). All items were rated on a 4-point scale with higher scores indicating greater satisfaction (alpha=.89). All questions were administered on a laptop computer with headphones, using audio-assisted interviewing.

We examined logs of the technology activity and calculated descriptive statistics for amount and frequency of use of each element, valence reported, and screen time.

The qualitative interview questions were guided by the EPMER [12] to gain an understanding of participants’ experience with the technology for increasing their emotion regulation skills at different points along the emotion regulation process. We also utilized aspects of the empirically derived unified theory of use and acceptance of technology (UTAUT) [51] to describe participants’ reactions of the technology components of the intervention. Masters-level interviewers trained in qualitative interviewing administered the interviews that lasted approximately 45 min. The interviews were transcribed and entered into Dedoose [52]. A “start” list of initial codes based on the UTAUT and the EPMER was created by the research team. The start list codes consisted of labels containing one to several words assigned to sections of the text that described that code. Guided by grounded theory [53]. the research team then met to review the codes, develop the codebook, apply the start list codes to the text, and create new codes based on emergent themes.

## Results

### Participants

There were 49 adolescent mothers enrolled in the intervention condition and their mean age was 18.54 years; 21% (10/49)of participants identified as white, 33% (16/49) black/African American; 42% (20/49) Latina, and 4% (3/49) other race/ethnicities. Approximately 40% (20/49) of adolescent mothers spent time in foster care in their lifetime; 62% (31/49) were held back a grade in school on at least one occasion. Most adolescent mothers had one child whose ages ranged from 0.2 months to 63 61 months (mean 16.4 months).

### Use of the Technology

#### Mobile App and Study Phone

Of the 49 participants assigned to the intervention condition, 4 were discharged before receiving a phone, 1 participant did not use the phone, and there were technology problems with 4 phones; therefore, we present data on 40 participants who had readable data.

#### Use of the Calm Mom Mobile App

Due to the varying amount of time adolescent mothers spent at the TLPs, participants had their study mobile phone between 15 and 316 days (mean 155.8; standard deviation [SD] 75.16) and in total spent 605.67 min (mean 14.77 [SD 14.39], range 0.62-76.34) using the app. Participants used at least one of the elements on the app on average; 44% of days they had the study mobile phone (SD 24.82).

### Mobile App Only Reports (Nightly and Self-Reports)

#### Nightly Reports

**Figure 6.**
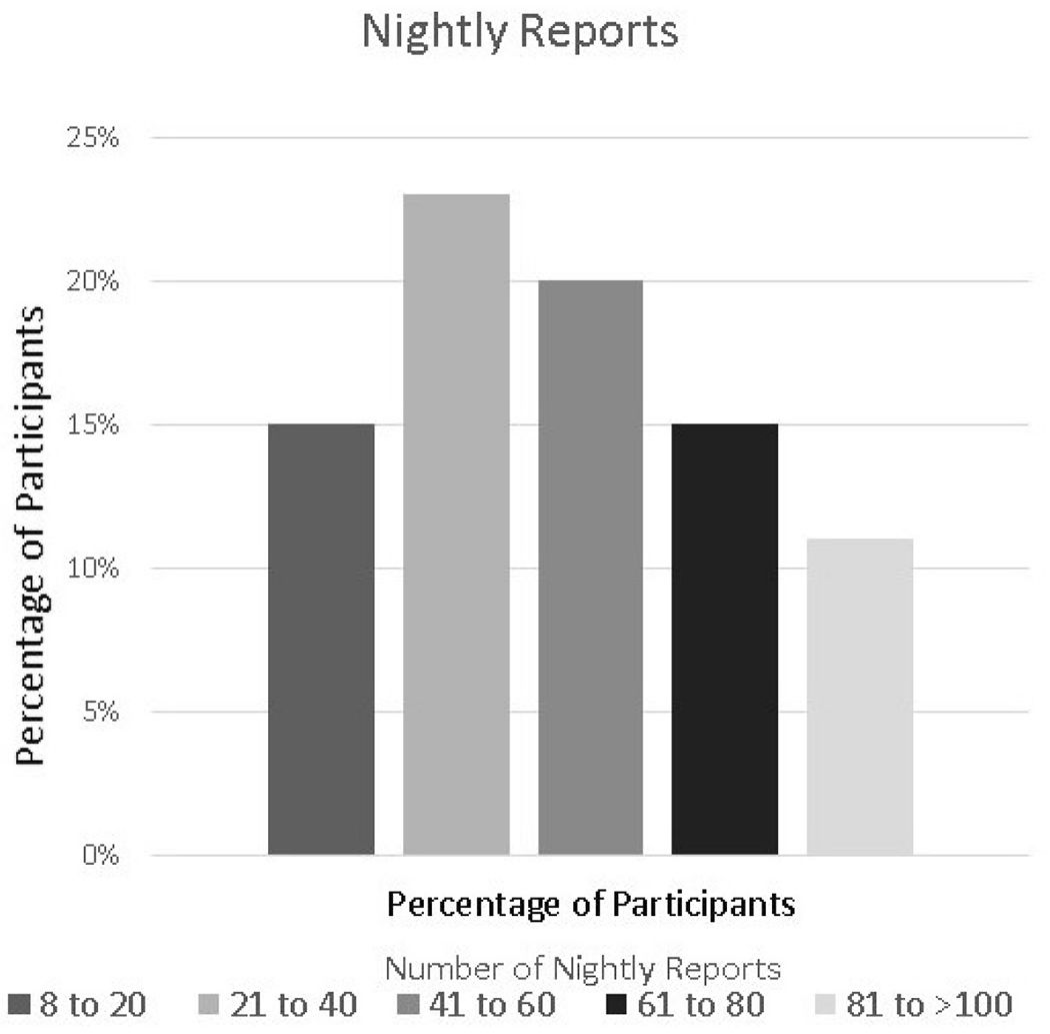
Distribution of the number of nightly reports.

Overall, participants answered approximately 40.00% (2555/6388) of the nightly messages (Figure 6). The average number of nightly reports per participant was 65 12 (SD 46.1, range 8-247). The overwhelming majority (88.00%, 2248/2555) of these reports were in the positive range with an average valence of 69.36 (SD 14.90) on the scale of 0 to 100 (50 and above is the positive range). The most frequently chosen valence was 50. Participants spent an average of 4.8 seconds (SD 4.03, range 1.36-18.26) making a nightly report.

#### Self-Reports

**Figure 7.**
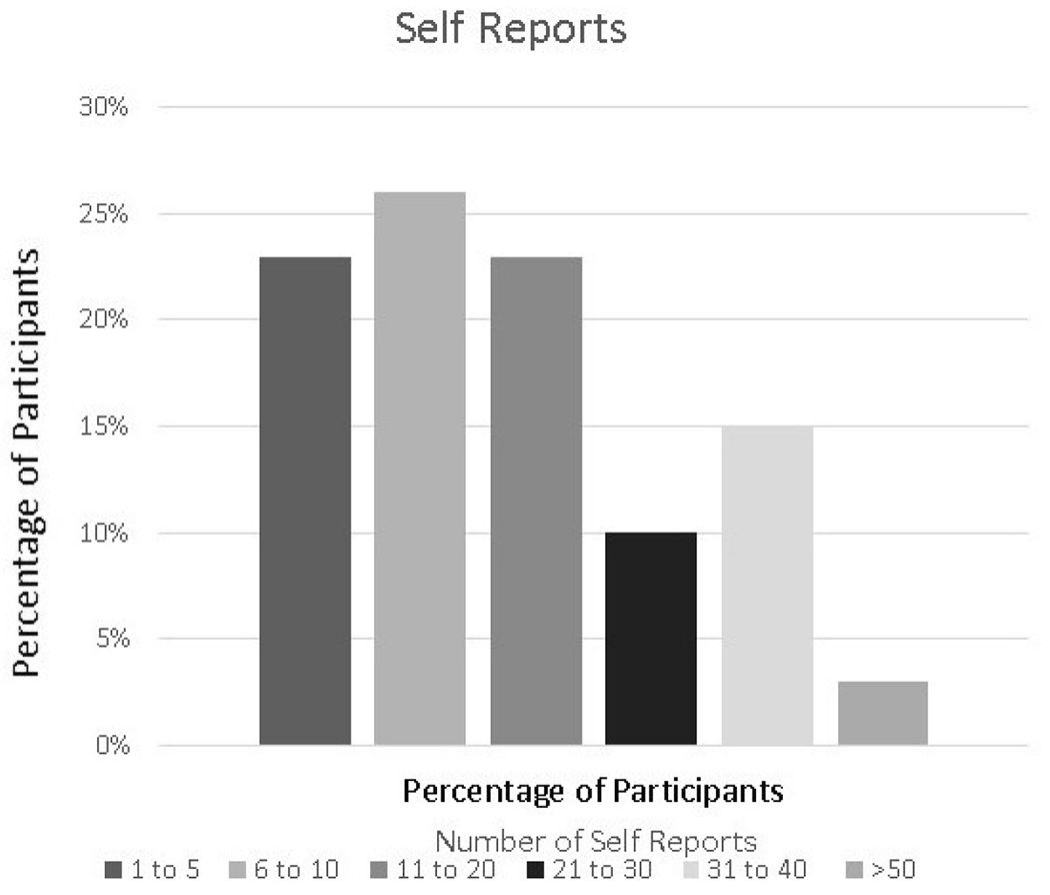
Distribution of the number of self reports.

In total, participants made 609 self-reports with 97% (39/40) of participants making at least one self-report (mean 16.4 [SD 15.08], range 1-75). Approximately half of the participants made 10 or fewer self-reports and the other half made between 11 and 40 self-reports (Figure 7). The mean valence reported was 47.30 (SD 16.50, range 11.70-88.89). There were more positively (335, 55.0%) than negatively valenced (274) self-reports (*P*=.01). Participants spent an average of 3.07 min (SD 4.91, range 7.71 seconds to 25.32 min) making a self-report.

We examined the mean valence of the nightly reports versus the self-reports; the mean valence of the nightly reports was significantly higher than those of the self-reports ((*t*_39_=2.94. *P*=.005).

### Sensorband Plus Mobile App

Of the 40 participants who received study phones, 7 participants left the TLP before they could receive the sensorband. Of these 33 participants, technical problems prevented 9 participants from using the band and 13 did not receive an alert. Therefore, only 11 out of the 33 (33%) participants who received a sensorband made one or more reports in response to sensorband alerts.

#### Sensorband Reports

Among these 11 participants, the number of reports varied between 1 and 71, with the vast majority (9/11, 77%) of participants making less than 20 reports. The average valence reported was slightly in the positive range (mean 57.23 [SD 11.2], range 43.95-81.43), with participants making significantly more positively valenced (192/218, 88.0%) than negatively valenced reports; however, the most frequently chosen valence was 50. Participants spent an average of 5.51 seconds (SD 9.4) making reports in response to sensorband alerts.

### Quantitative Acceptability Data

The overall average rating on the acceptability scale was 3.55 on a 4-point scale, which ranged from 3.32 to 3.85 (Table 1). Specifically, 75% of participants (N=40) were “very” satisfied with the help they received from the blended intervention and 18% were somewhat satisfied. Participants reported using skills learned in the in-person sessions that were reinforced on the mobile app. Over 87% of participants reported using “cool thoughts and good moves” and over 82% used “Freeze, breathe, and choose,” “very,” or “somewhat” often. Over 94% and 92%, respectively, indicted that the sensorband and mobile app helped them manage their stress and deal with their child “very much” or “somewhat.”

### Qualitative Results

#### Performance Expectancy

Performance expectancy refers to the degree to which an individual believes that the use of the technology will be helpful [51]. Within this construct, we identified 2 overall themes relating to the impact of the Calm Mom technology: (1) overall perceived benefits of the technology and (2) enhancing identification of emotions and emotion regulation strategies.

##### Overall Perceived Benefits of the Calm Mom Technology

Participants felt that the general content of the app was highly related to their lives as adolescents and new mothers. One noted that she liked the name *“Calm Mom”* because:

*…it makes me want to use it because I want to be a calm mom.* [P235]

Another explained:

*…it was cool to just like connect with the phone…it’ll put like, how are you feeling about your boyfriend or love-wise, school-wise, stuff like that.* [P230]

##### Relieving Feelings of Isolation and Obtaining Support and Guidance

A majority of participants noted that the app was very accessible, and several indicated that just having the technology made them feel less alone and genuinely cared for:

*…well at least somebody is asking me about how my day went or how I feel today…It made me feel differently because it actually felt like, like somebody was listening and they at least give you feedback*. [P249]

All participants commented that the affirmational messages and the skills-based “cool thoughts” and “good moves” messages were particularly helpful:

*It would give me some advice, things like, you know, “just take one step at a time.”* [P242]

### Enhancing Identification of Emotions and Emotion Regulation Strategies

Participants believed that engaging with the technology supported their emotional regulatory skills at several points along the process, particularly in prompting self-monitoring, identifying and appraising their emotions, and encouraging self-reflection (Table 2). With one exception, all of the adolescent mothers characterized their interaction with the app as giving them an opportunity to pause for a moment, which assisted them in deploying their attention to their current feelings and subsequently, strategies for coping:

*…I just liked that because it made me stop and really think and just calm down*. [P231]

The messages also helped participants to adopt an attitude of mindfulness that, in turn, helped to bolster their mood or reduce their feelings of stress:

*…like if I was having a bad day it was like…oh it’s okay…this won’t last forever*. [P226]

Additionally, labeling and scaling their feelings on the app increased self-knowledge:

*…just doing the little mirror helped me realize how stressed I really am…at what level of stress I was at…* [P234]

For some, pausing to reflect on their feelings appeared to increase their motivation to regulate their emotions and use one of the skills taught in the in-person sessions:

Like it says, “freeze, breathe and choose” because you can actually stop what you are doing, think about it, and choose whether it is the right way or the wrong way to deal with it.

#### Individual Preferences

There were considerable individual preferences as some participants preferred the videos because they could relate to the character (“they were like girls who looked like they could be here,” P235), whereas others favored the written messages. Several participants commented that sometimes the messages were repetitive or not related to their current stressor, but P228 noted, “*some of them weren’t really on point but they had a lot to do with it.”* P244 indicated that sometimes she gets a “*perfect message and it really, really helps.“*

The initial “bank” of messages for valences in the positive range was very small, and several adolescent mothers at the start of the study complained that these messages were very repetitive; therefore, midway through the study, we increased the number of messages that were provided when participants selected an emotional valence in the positive range.

#### Nightly Reports

Overall, the nightly “push-in” reports were the most frequent reports made by participants who used these to reflect on their mood after their children were asleep:

*It was good because my kids were sleeping so I got to think about it…and take me out a little bit of my anger*. [P249]

For P233, the regularity of the nightly reports helped to reinforce her ability to self-regulate:

Um just so you can have something to read every day, something to remind you to keep calm and think about what you do and breathe…

On the other hand, P228 noted that although the nightly reports were helpful when she was angry, they were not useful when her mood was positive:

When I’m just chill and it popped up, it got kind of annoying…it wasn’t really relevant to anything but when I was actually mad and it popped up, it was a good reminder.

Several participants reflected on the nightly reports in relation to putting their children to bed, a typically challenging time for parents of young children.

P234: And sometimes it would be rough for him to fall asleep. Or he would be cranky…so if my phone started buzzing for the evening report I would put in…I was feeling bad because the baby was cranky and…um…it would say something to calm me so I would take a deep breath and then just relax and ignore. It’s not really so much ignore the fact that he was cranky but ignore it enough for him to calm down and fall asleep.

Interviewer: Do you think that changed the situation?

P234: If I hadn’t gotten the report I would’ve probably been a little bit more stressed…I think it kind of like calmed him down a bit because he realized that he wasn’t getting a reaction from me as he was being so cranky and he just ended up falling asleep. ’Cause usually…like I will take him out of the bed and let him stay up longer…

In this instance, the app supported the participant’s ability to engage in more mindful parenting as it drew her attention away from her son’s “crankiness” to an awareness of her own feelings in the moment. This mindful attending then allowed her to momentarily accept and tolerate her son’s mood and refrain from taking him out of the crib, enabling him to fall asleep faster. She also keenly observed that the nightly report aided both her and her son.

#### Self-Reports

Many of the themes evidenced in the nightly reports were echoed in participants’ experiences engaging in self-reports. The main difference was that instead of a “push-in” reminder, self-reporting was purely volitional where participants could actively select to engage with the app.

*It just gave me a moment that I could use for myself*. [P236]

*…it gave me self-control because I could basically get them whenever I wanted to and had access to them all the time*. [P235]

Moreover, mothers were able to hold a mental representation of the app and its benefits when cued by stressful situations.

*…because if you are haviing a bad day you can be like, “I need a Calm Mom.”* [P230]

She describes a particular instance when the app helped her relieve the tension associated with the many demands on her as a mother:

Yeah, because I had to go to my doctor’s appointment, got my eyes checked…and then had to come home and get him [child] to eat, and then go back for his doctor’s appointment, and then come back, and then put him to bed and so that was a stressful long day and I was like, let me make a report.

Self-reporting was often cited by participants as a self-caring activity as participants engaged in self-reporting when they were feeling, “alone,” “down,” or “bored.” Additionally, the self-report was useful during challenging situations with children, family members, peers, or romantic partners. P233 recounted how after completing a self-report she was able to use self-talk to plan a more adaptive behavior in the midst of having a disagreement with another resident:

It’s helpful because I don’t want to get myself in trouble or come at somebody the wrong way because I’m because I’m angry…but if I read it I would be like…maybe I should calm down and talk to them maturely and not yell.

Finally, self-reporting was also used as a strategy to engage in more effective parenting:

P249: And I would just make a self-report if my son wasn’t cooperating with me or my daughter was being, you know, disrespectful.

Interviewer: And did it change how you responded to your child?

P249: Yeah, it did, I stopped and thought before I would say anything or…responded to what they did. It helped me basically control the situation without yelling or other stuff; there were alternatives.

#### Sensorband

As a result of technical challenges with the sensorband, fewer participants received notifications on their smartphones in response to heightened levels of EDA. However, for participants who received the alerts, many reported that the notification caused them to stop and pay attention to their actions.

*…it would send those messages to my phone and some of the time it did help because um, I don’t know, sometimes when I get stressed out I really don’t think about what I’m doing so it’s like having those little reminders it really helped sometimes.* [P228]

A few participants expressed surprise that the sensorband could detect that they were feeling stressed.

*I was wearing this sensorband that was feeling [my] mood, just like, you know, having a stressful day…it actually chimed, it actually knows*. [P230]

Additionally, P242 explained that the alerting made her feel cared for:

…even with no one being there, I still felt like someone was there…

A number of participants related that alerting of increased EDA often occurred when they were involved in an argument. For P231, the alert confirmed the intensity of her feelings and assisted in modifying her response:

And as soon as the argument was getting to a point where I was actually getting mad…it just started going off and that’s when, you know, I paused for a second while she just kept running her mouth screaming and I looked towards my phone, read what it said to me, and I just froze and I was like, “look, you’re right…let’s just leave it at that.” And I just walked away and did what I needed to do for the day. I wasn’t really thinking about my actions until I saw it, like I felt it and that’s when I was like…alright…and it actually calmed me down.

Alerts in response to escalating EDA also assisted participants to attend to and reappraise their stress and allow them to engage in situation selection by using parenting skills learned in the intervention. P230 describes a time when she missed her bus and would get home late to cook dinner for her son:

…at that time I was stressful, like on the bus I was just like, “Oh my goodness, am I going to make it? He is going to come home angry…there s no food…” And then after it beeped I just thought about it and was like, okay, just do what I have to do now for A [son]…let me just get started. Let me give him something to do, or do something with him while I’m cooking.

At times, the sensorband alert alone acted as a cue to draw participants’ attention to their emotion and modulate their response without engaging with the app. P243 explained that she tended to disregard the content of the app but engaged in self-talk after hearing the alert that her EDA had risen:

I just paid more attention to you know when the phone going off…notice that I was feeling angry and then I would know, “alright, I need to calm down.”

She also indicated that at other times the alert was not helpful because:

All it did was told me which I already knew…that I was getting upset.

### Effort Expectancy

Effort expectancy refers to the degree of ease of using the technology. All participants found the app easy to use and navigate. By comparison, participants had mixed experiences with the sensorband.

### Functionality of the Sensorband

The greatest challenge reported by participants involved difficulty knowing when the band was charged and working properly (“I thought it was charging but it would just die,” P233). Additionally, some participants felt as if the band was too sensitive (“I felt as if I was getting Calm Mom assessments every 5 minutes,” P228). Several participants expressed disappointment that they did not receive an alert at times when they were stressed:

*I just want to know when I’m stressing and when I’m not*. [P233]

Moreover, wearing the sensorband and not knowing if it was working diminished participants’ enthusiasm for using it.

### Facilitating Conditions

Facilitating conditions refers to the compatibility between individuals’ lives and the use of the technology. We include comfort and fit of the sensor band in this construct.

#### Challenges Integrating Use of the Sensorband Into Their Daily Routines

Although most participants used the study smartphone as their main phone, managing 2 devices was challenging:

*I don’t have any time. I’m always like, doing something and then when I get home I’m always doing something, for my son or like my boyfriend or something…* [P228]

The transient lifestyle of some adolescent mothers also presented a challenge:

*I didn’t wear the band as much as I should have because I was moving from shelter to shelter and I did, at one point, misplace the band*. [P236]

#### Comfort, Fit, and Appearance

Participants were mixed about the level of comfort, although some found it comfortable and often forgot they were wearing it:

*Once I put it on in the morning, I didn’t really notice it.* [P235]

Others found it “bulky,” “itchy,” or “too big.”

#### Social Issues

Several participants indicated that they were asked if the sensorband was a legal monitoring or probation bracelet when they wore it outside of the TLP, which was described as bothersome and sometimes “embarrassing.” Others described that wearing the sensorband generated questions from others:

*Um, they thought it was interesting and kind of…a few people wanted to participate in it…and others were like…oh, that’s pretty cool…* [P231]

## Discussion

### Principal Findings

We found a high degree of acceptability among homeless adolescent mothers for the technology components of a blended intervention designed to increase emotional regulation. Using both the quantitative and qualitative data, we found that participants highly valued the accessibility of the Calm Mom app both alone and in combination with the sensorband. For many adolescent mothers, the app became an integral part of the ways in which they dealt with heightened emotions in stress-inducing situations. Qualitative findings indicated that engagement with Calm Mom elements increased adolescent mothers’ ability to increase their mindful attention to their experiences in the present moment, which is an integral aspect of the ability to adaptively regulate emotions and a primary goal of the parenting intervention.

The Calm Mom technology assisted adolescent mothers to effectively regulate their emotions in theoretically meaningful ways and marks a meaningful contribution to the mHealth literature, which has been somewhat limited in the application of behavioral theory to technological interventions [54]. Adolescent mothers reported that use of the app—and for a smaller proportion of participants, in combination with the sensorband—increased their identification and understanding of their emotions in a variety of stressful situations with their children, peers, and family, which in turn helped them engage in more adaptive emotion regulation and behavioral strategies.

Independent of the sensorband, we observed high response rates to both the “push-in” nightly reports and the “pull” self-reports, and there was a considerable range of the number of nightly and self-reports. As expected, and in line with other research [55,56], there were fewer self-reports relative to the nightly “push-in” reports, which prompted adolescent mothers at the same time every evening and involved a very brief, low-demand response along with a well-liked affirmational message. Additionally, the average screen time for the nightly reports was shorter than the self-reports, which involved more content. As detailed in the qualitative data, for many adolescent mothers, the nightly reports arrived at an opportune time when they had a quiet moment to reflect on their day or, in some cases, when putting their child to bed. Regarding the self-reports, qualitative analysis revealed that adolescent mothers were able to maintain a mental representation of the app as a tool for helping them cope with their many daily stressors, an important facilitator of engagement with the app. In this way, the technology served as a “virtual holding environment” [57], thereby scaffolding adolescent mothers’ attempts at regulating their emotions. Yet, the fact that many of the adolescent mothers reported relying on the app for comfort and to ease their loneliness highlights the acute vulnerability of this population of young mothers who are homeless and straggling to meet numerous demands.

Surprisingly, for all elements of the Calm Mom technology, the average valence selected on the feeling scale was in the positive range. This may be due to the design of the “How are you feeling” screen where the default choice on the slide bar was “50” and considered in the positive range. In future research, we will use other ways of eliciting emotion such as the circumplex model [58].

Although we encountered considerable technology challenges with the sensorband, the smaller number of participants who experienced the full capability of the Calm Mom technology (app plus sensorband) reported high levels of acceptability and notably experienced benefits consonant with the theoretical model of the intervention. Importantly, participants who received alerts via the sensorband expressed that they were typically emotionally aroused when they received the alert. Although previous research of wearable sensors for the measurement of EDA has been limited to small, proof-of-concept studies [26,27,59], this is one of the few studies to deliver “just-in-time” behavioral intervention strategies over an extended period of time to capture real-life stressful situations among a highly vulnerable population of adolescent mothers.

A comprehensive understanding of the needs, challenges, and life circumstances of the target population is vital for designing any behavioral intervention and may be particularly important for mHealth interventions as participants receive the intervention during the course of their daily lives [60], outside of their interaction with intervention facilitators. mHealth technology can be experienced as demanding [59,61], and we did not want Calm Mom to distract adolescent mothers from their children or tax the cognitive and emotional capacity of these highly overburdened young mothers. Thus, providing adolescent mothers with a variety of ways to engage with the technology and varying the content of the app emerged as strengths of our design and helped to meet individual preferences for engagement [62].

In this acceptability study, we did not aim for “compliance” per se; rather, we sought to understand adolescent mothers’ receptivity to the technology with respect to the frequency, timing, mode (nightly or self-reports or sensorband alerts), media (eg, video vs text), and content, particularly the reinforcement of skills taught in the in-person intervention [63]. Our findings underscore the need to design mHealth interventions that use more refined algorithmic tailoring over time based on participants’ responses and levels of stress and preferences [17]. For instance, for some participants, receiving an alert in a stressful moment was opportune, whereas for others, it was perceived as irritating. Thus, in future studies, we plan to refine decision rules that adapt to individuals’ dynamically changing states and preferences to enhance personalization and capitalize on both states of vulnerability and opportunity [17]. For example, alerts provided when stress levels are just beginning to increase may be a more ideal window of time for some participants. Importantly, the provision of support must augment rather than disrupt individuals’ existing, autonomous effective coping strategies.

The EPMER was extremely useful for understanding the theoretically derived mechanisms by which adolescent mothers used the technology and the ways the technology was able to augment in-person, provider-delivered sessions. The qualitative interviews revealed that many adolescent mothers valued the way specific skills learned in the in-person sessions were translated onto the app, helping to bridge their experience of the in-person sessions in their daily life. In future research, we will focus on specifying the exact mechanisms by which the in-person and technology components complement one another.

## Limitations and Future Directions

The primary limitation of the study was the small number of participants who were able use the sensorband due to technological challenges that are typical when new technologies are tested in the field. Similar to other mHealth studies that have collected affective data using objective, longitudinal methods [64], we encountered operating system problems as well as issues related to the form and functioning of the sensorband. Although we piloted the sensorband over a short period of time with a small number of participants from the target population [48], over the course of the study, major upgrades occurred in the operating systems of both Android and Bluetooth, which resulted in the loss of connectivity for a number of participants. Furthermore, participants may not have always worn the sensorband correctly, which prevented transmission of EDA; therefore, in future research, utilizing a “run-in” period with the sensorband may be warranted. Although these issues will be addressed in future studies, the current limitations of the technology may impede scalability. However, sensor technology is advancing at a rapid pace, and the reliability and design of devices for measuring ambulatory EDA and other physiological measure of stress continue to improve [65–68]. Future studies will benefit from these advances coupled with the increasing popularity of wearable activity trackers and smart watches, which will help to reduce social issues some participants encountered when wearing the sensorband. Finally, determining the correct “dose” regarding the timing and duration of technological support in conjunction with provider-delivered intervention is an important avenue of future research.

Despite the challenges we encountered, our findings suggest a high level of enthusiasm and acceptability for the Calm Mom technology and patterns of use consistent with the underlying theoretical model, all of which will be harnessed in future studies as the technology improves.

## Supplementary Material

Appendix

## Figures and Tables

**Table 1.** Acceptability questionnaire with a 4-point acceptability scale (N=40).

Survey item^a^	Mean (SD)
How satisfied are you with the help that you received from the Power Source Parenting program?	3.74 (0.60)
How well did Power Source Parenting program help you deal more effectively with parenting problems?	3.65 (0.63)
How much did you use the concept of Cool thoughts/good moves outside of group?	3.50 (0.77)
How much did you use the concept of freeze, breathe, and choose outside of group?	3.32 (0.94)
How well did the Power Source Parenting group help you deal with your stress?	3.33 (0.96)
How much did you like using the band and the smartphone?	3.85 (0.36)
How helpful was the band and smartphone in managing your stress?	3.57 (0.65)
How helpful was the band and smartphone to you for dealing with your child?	3.47 (0.65)

a Derived from the client satisfaction questionnaire [50].

**Table 2.** Examples of Qualitative Responses of Emotion Regulation Strategies by Modes of Engagement with Calm Mom

Mode of Engagement and qualitative response	Emotional Regulation Strategies
**Nightly report**	
“reminded me everyday”	• Attentional deployment
“something to remind you to keep calm and think about what you do and breathe”	• Attentional deployment • Cognitive change • Response modulation
“I liked waiting for the motivational message to come in…help me just be relaxed and calm”	• Attentional deployment • Cognitive change • Response modulation
“it was good so sometimes you don’t go to bed angry or upset”	• Attentional deployment • Cognitive change • Response modulation
**Self-report**	
“gave me a moment that I could use for myself’	• Situation selection
“if you are having a bad day, you can be like, ‘I need a Calm Mom’”	• Situation modification
“I could just sit down and take time to do the report”	• Attentional deployment
“would make me feel better about myself so I did not have to be upset the whole day”	• Cognitive change • Response modulation
“gave me self-control ‘cause I could basically get them whenever I wanted”	• Situation selection • Situation modification
“gave me a moment I could think”	• Attentional deployment
“helped me notice feelings”	• Attentional deployment
“I stopped and thought before I would say anything”	• Attentional deployment
“helped me control the situation without yelling or other stuff, there were alternatives”	• Cognitive change • Response modulation
**Sensor Band Report**	
“it does not put you in a chaotic situation like you would if you didn’t really think about it”	• Situation modification
“helps you stop, think, and breathe”	• Attentional deployment • Cognitive change • Response modulation
“I was yelling but then I went upstairs and calmed down and them came back down to apologize”	• Situation modification • Attentional deployment • Cognitive change • Response modulation
“so I chilled out and didn’t talk to him for like three days and that was good”	• Situation modification
“so I flip out…and it sent a thing to my phone about your baby feeling what you feel and made me realize that it is not about me anymore”	• Attentional deployment • Cognitive change
“made me meditate to calm down”	• Response modification
“even with no one being there I still felt like someone was there”	• Cognitive change
“I paused fora second…read what it said to me…and I was like, ‘look, you are right, let’s just leave it at that’”	• Attentional deployment • Cognitive change • Response modulation

## Abbreviations

CBT: cognitive behavioral therapy
EDA: electrodennal activity
EPMER: extended process model of emotion regulation
IRB: institutional review board
mHealth: mobile health
TLP: transitional living program
UTAUT: unified theory of use and acceptance of technology
