# Supplementary figures and images for: Theoretically-Based Emotion Regulation Strategies Using a Mobile App and Wearable Sensor Among Homeless Adolescent Mothers: Acceptability and Feasibility Study
Source: JMIR Pediatr Parent. Author manuscript; Available in PMC 2019 Jan 9. (PMC6326370; doi:10.2196/pediatrics.9037)

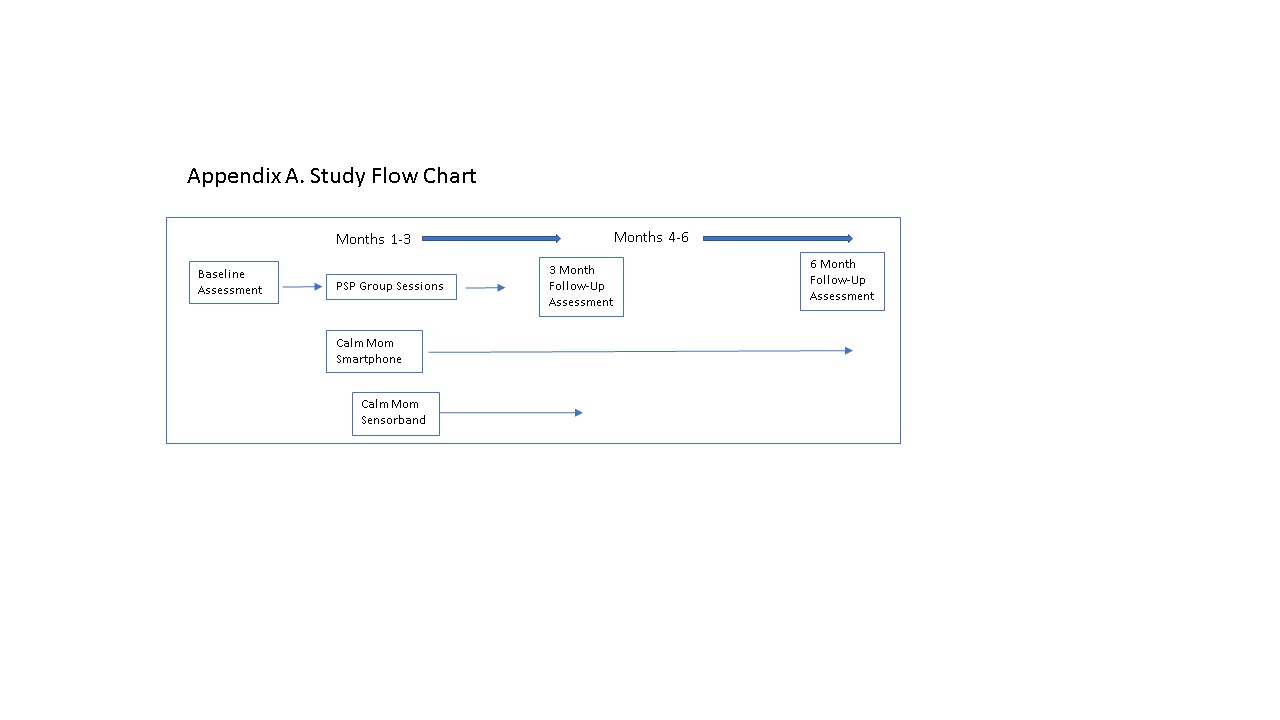

Supplement: Appendix [file NIHMS994040-supplement-Appendix.jpg]
